# A computational approach for the identification of key genes and biological pathways of chronic lung diseases: a systems biology approach

**DOI:** 10.1186/s12920-023-01596-7

**Published:** 2023-07-08

**Authors:** Hadi Rezaeeyan, B. Fatemeh Nobakht M. Gh, Masoud Arabfard

**Affiliations:** Chemical Injuries Research Center, Systems Biology and Poisonings Institute, Baqiyatallah University of Medical Sciences, Tehran, Iran

**Keywords:** Systems biology, COPD, IPF, Asthma, Protein–protein interaction network, Mustard lung disease

## Abstract

**Background:**

Chronic lung diseases are characterized by impaired lung function. Given that many diseases have shared clinical symptoms and pathogenesis, identifying shared pathogenesis can help the design of preventive and therapeutic strategies. This study aimed to evaluate the proteins and pathways of chronic obstructive pulmonary disease (COPD), asthma, idiopathic pulmonary fibrosis (IPF), and mustard lung disease (MLD).

**Methods and results:**

After collecting the data and determining the gene list of each disease, gene expression changes were examined in comparison to healthy individuals. Protein–protein interaction (PPI) and pathway enrichment analysis were used to evaluate genes and shared pathways of the four diseases. There were 22 shared genes, including ACTB, AHSG, ALB, APO, A1, APO C3, FTH1, GAPDH, GC, GSTP1, HP, HSPB1, IGKC, KRT10, KRT9, LCN1, PSMA2, RBP4, 100A8, S100A9, TF, and UBE2N. The major biological pathways in which these genes are involved are inflammatory pathways. Some of these genes activate different pathways in each disease, leading to the induction or inhibition of inflammation.

**Conclusion:**

Identification of the genes and shared pathways of diseases can contribute to identifying pathogenesis pathways and designing preventive and therapeutic strategies.

**Supplementary Information:**

The online version contains supplementary material available at 10.1186/s12920-023-01596-7.

## Introduction

Lung is a basic organ regulating and maintaining the function of the respiratory tract [1, 2]. Recent data have shown that respiratory disorders affect many people in the USA (35 million people, most of whom deal with asthma and chronic obstructive pulmonary disease (COPD)). This disorder leads to various diseases in patients, which are associated with many deaths [3]. A rise in the mortality of patients due to respiratory disorders is a major global challenge that necessitates the identification of the pathogenesis of diseases and the use of preventive and therapeutic strategies [4].

There is a wide range of chronic lung diseases which overlap in terms of clinical symptoms and are difficult to distinguish [5]. In addition, disease pathogenesis is multifactorial, and the main cause of chronic lung disease has not yet been identified. Some chronic lung diseases, including COPD, asthma, idiopathic pulmonary fibrosis (IPF), and mustard lung disease (MLD) have shared clinical symptoms such as mucus secretion, cough, impaired lung function, and dyspnea.

Recent evidence suggests that inflammation is shared in all the four mentioned diseases, leading to disease progression. The chemotaxis of immune cells and their cytokine production are the other factors in the pathogenesis of diseases [1, 6–8]. However, in COPD and MLD, inflammation stimulates the immune system and coagulation factors’ secretion, which ultimately causes coagulation in patients [9, 10]. Inflammation in asthma causes T cells to differentiate into T helper2 (Th2), and eventually produces a series of cytokines, including IL-5 and IL-13, which lead to mucus production and secretion in patients [11]. In IPF, inflammation leads to TGF-β production, a cytokine that causes pulmonary fibrosis in patients [12].

Protein–protein interaction (PPI) handles a wide range of genes and proteins and creates a network between them in cellular communication [13]. In fact, gene ontology (GO) shows the molecular pathways, cellular functions, and biological processes of each gene and protein in a disease. In other words, PPI depicts the network created between genes and proteins, as well as the commonalities between them in cellular communication [14–16].

Lung involvement is a shared pathogenesis and identical genes and molecular pathways are involved in the pathogenesis of the four diseases. Gene study has been evaluated individually in chronic lung diseases, and no study has assessed the expression of shared genes and proteins in several diseases. Thus, the present study evaluated the association between genes and shared proteins in COPD, asthma, IPF, and MLD. Biological pathways, cellular components, and molecular functions of proteins were also examined.

## Materials and methods

### Data collection

PubMed, Scopus, ISI Web of Science, and Cochrane databases were used to collect the data. All relevant studies were extracted and evaluated. Only case–control studies were included. Protein expression changes (increase or decrease) were measured compared to the control group. All the proteins were selected from proteomics studies. After data extraction, duplicates and missing data were deleted.

**Fig. 1:**
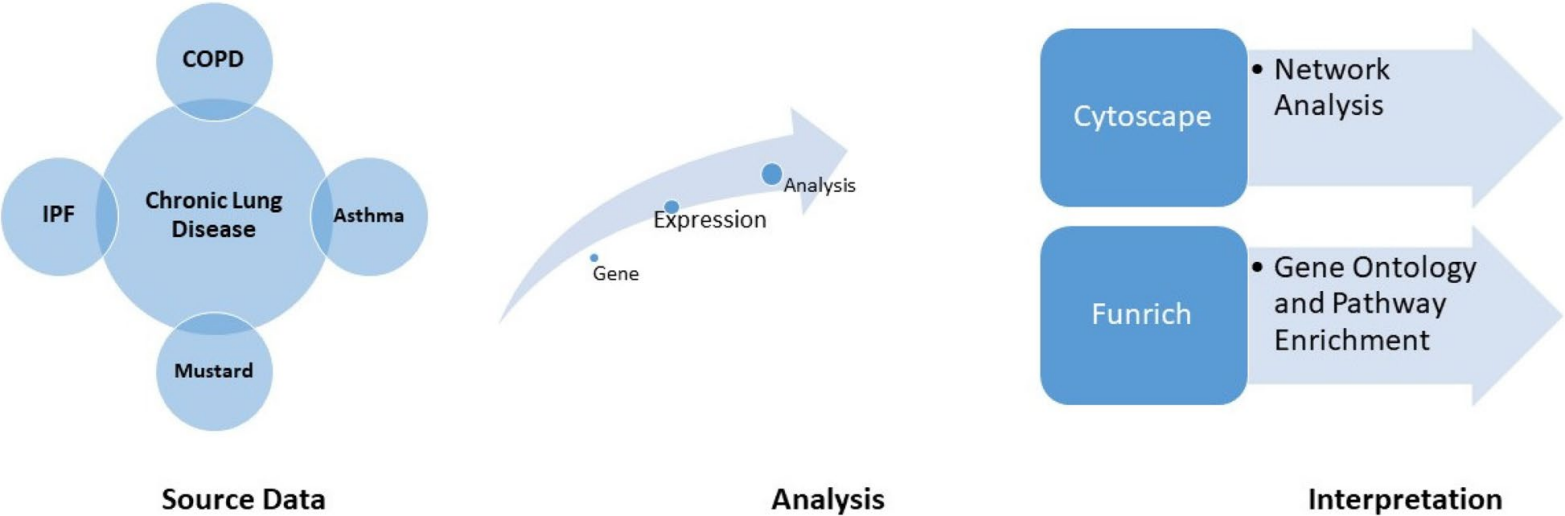
Workflow of data collection and analysis of genes and pathways

**Fig. 2:**
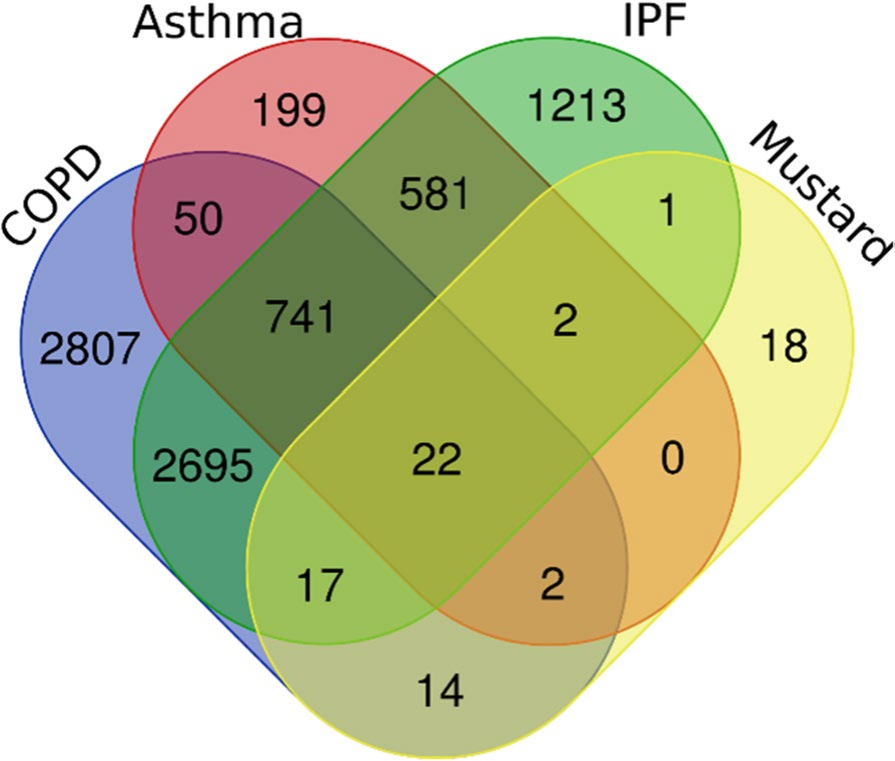
Scheme of total protein in COPD, asthma, IPF, and MLD

After identifying the relevant articles and extracting the studied genes, dysregulation was found in the expression of 13,940 genes in COPD, 2700 genes in asthma, 6686 genes in IPF, and 104 genes in MLD. A list of all the genes with fold change, *p*-value, and FDR is presented in Supplementary S1. For some genes, *p*-value, fold change, or FDR were not reported. After removal of duplicates, 6,348 genes in COPD, 1,597 genes in asthma, 5,272 genes in IPF, and 76 genes in MLD had up- or down-regulation. Then, using Genes Set, enrichment pathways involved in GO were identified (Fig. 1). The pathways with the highest score in which more genes were involved were then selected (Fig. 2).

Based on the screened studies, proteins identified as having different expressions under varying processing conditions within each study were also considered. The studies included COPD (*n* = 49), asthma (*n* = 23), IPF (*n* = 25), and MLD (*n* = 6). Furthermore, some studies only listed the proteins that showed expression changes without indicating the extent of these changes. Therefore, in this study, no threshold level for protein expression variations was established.

### Protein Interaction Network

To draw the protein interaction network, all the data were combined in a database; then, https://string-db.org/ was used for analysis. Physical and molecular interactions between proteins were identified and extracted. Finally, all data, networks and relationships between proteins were entered to Cytoscape (https://cytoscape.org/). Finally, the hub genes with the highest degree were identified using network analyzer packages.

### Shared gene and pathway analysis

**Fig. 3:**
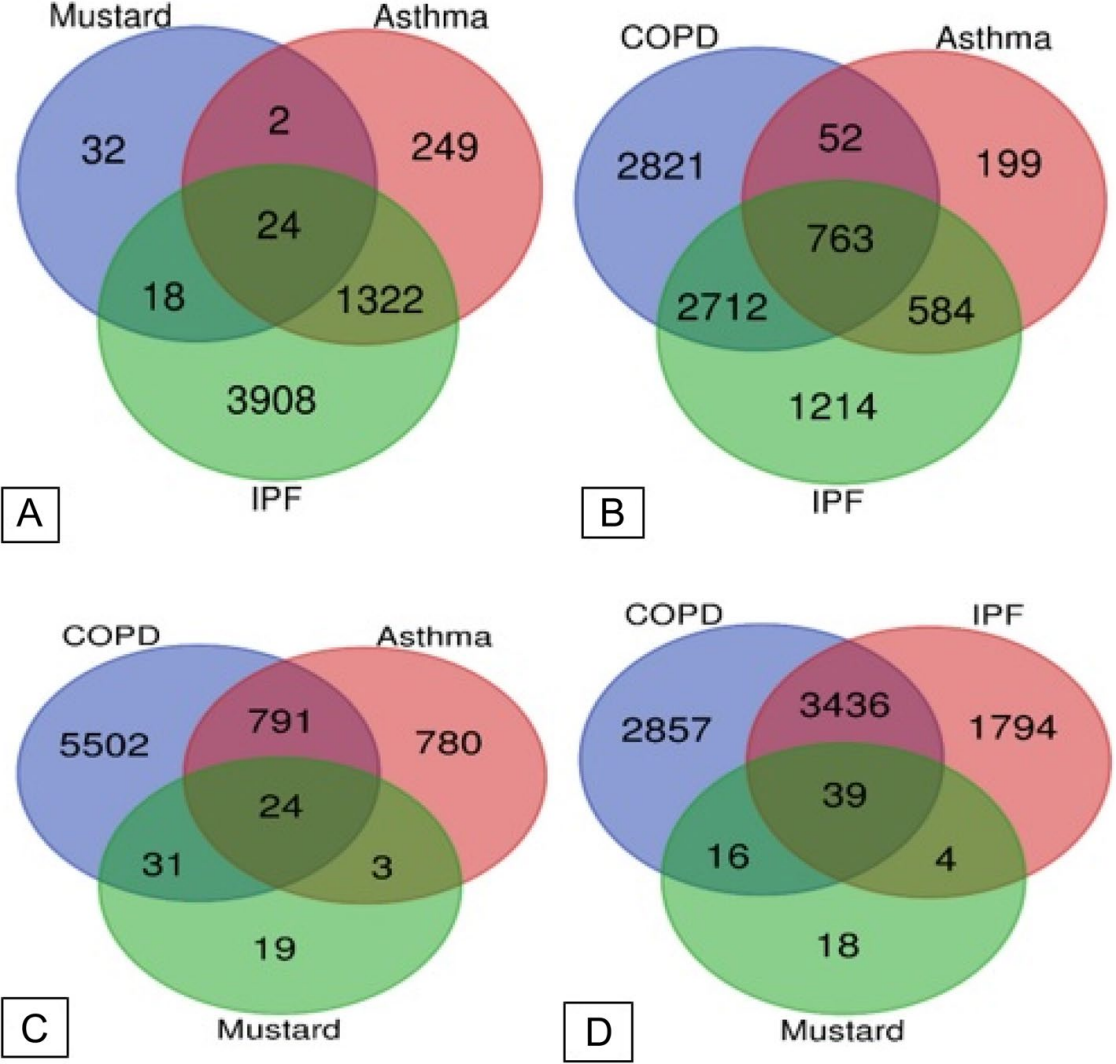
Scheme of the number proteins shared by the diseases. **A** number of proteins between MLD, asthma and IPF, **B** number of proteins between COPD, asthma and IPF, **C** number of proteins between COPD, asthma and MLD, **D** number of proteins between IPF, COPD and MLD

**Fig. 4:**
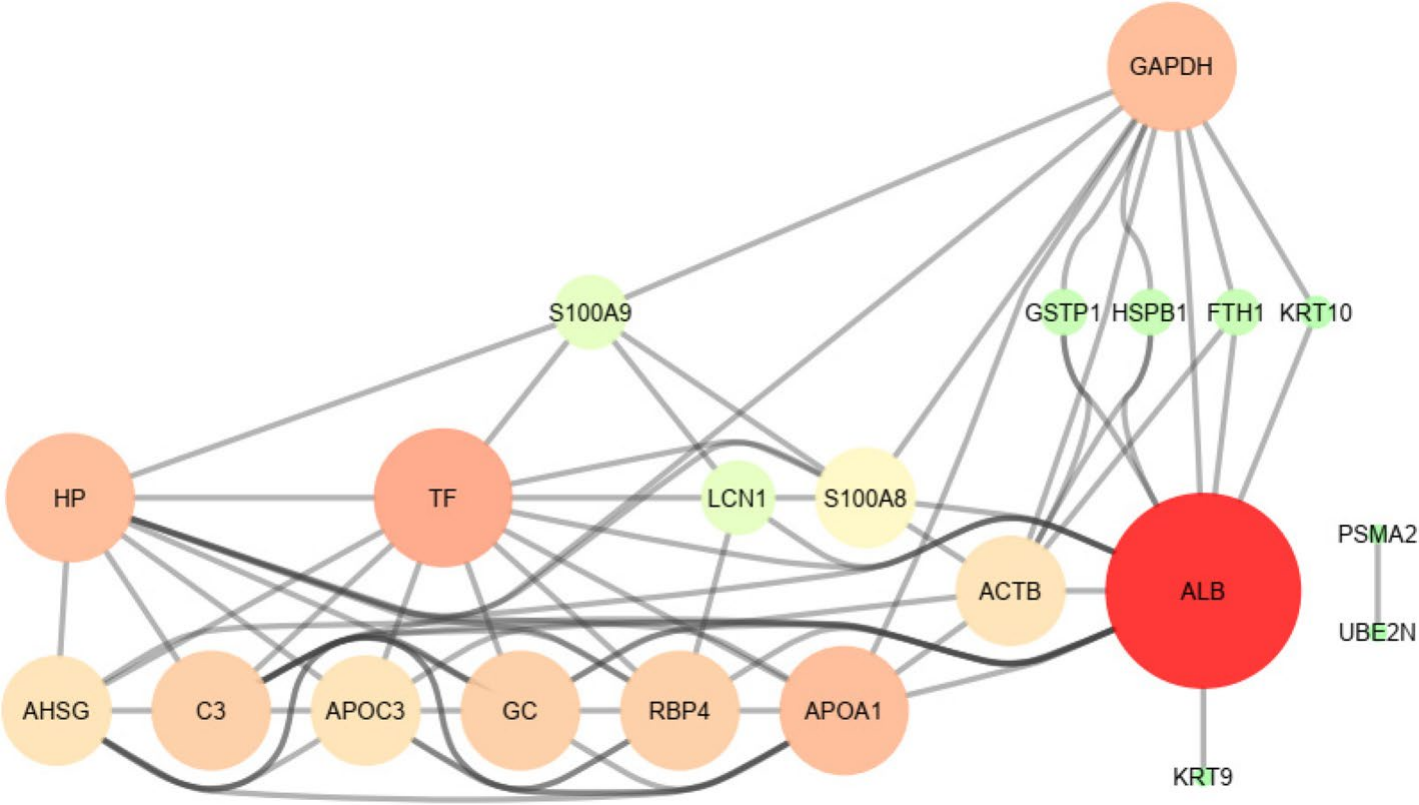
Scheme of 22 proteins shared by COPD, asthma, IPF and MLD. The nodes' size and color indicate the number of interactions they have with other proteins. Hence, a node with a larger size and a redder color indicates more interactions and points with other proteins, whereas a smaller and greener indicate a lower degree of that node

Shared genes of the mentioned diseases were examined. Diseases were also classified into three and two groups, and the shared genes were evaluated. Also, enrichment analysis was performed for the diseases individually and together (Figs. 3 and 4).

### Enrichment analysis

FunRich (http://www.funrich.org) was used to evaluate the gene ontology enrichments of the listed genes. These data were used to evaluate biological and molecular similarities of the genes.

## Results

This study examined shared genes and their associated biological pathways among four chronic lung diseases (COPD, IPF, asthma, and MLD) using PPI networks and enrichment analysis. In addition, changes in gene expression in each disease were identified separately. The role of genes in cellular metabolism and the biological processes involved in lung pathogenesis were also investigated.

### Similarity analysis for disease genes

The genes shared by the diseases were examined. For this purpose, genes shared by the diseases were studied in two and three groups. In the ternary group, there were 763, 24, 24, and 39 genes shared by COPD-asthma-IPF, COPD-asthma-MLD, asthma-IPF-MLD and IPF-MLD-COPD, respectively. Genes shared between the three diseases are shown in a Venn diagram (Fig. 3). In the pair groups, there were 815, 3475, 55, 1346, 26, and 42 genes shared between COPD-asthma, COPD-IPF, COPD-MLD, asthma-IPF, asthma-MLD and IPF-MLD, respectively. Moreover, 22 genes were shared by the four diseases (Fig. 4). Table 1 lists these 22 genes along with their up- or down-regulation in each disease. Using Enrichr, interactions between unique proteins in each disease were designed. Further evaluations showed that the GRB2 gene in both COPD and MLD and the ESR1 and IRF7 genes in asthma and IPF had the highest association with the other genes, respectively (Supplementary file S2). The names of unique proteins in each disease, along with their interaction information with other proteins, are reported in Supplementary file S3.

**Table 1:** Summary of up and down-regulation of 22 proteins shared between COPD, asthma, IPF and MLD

**Gene Name**	**Symbol**	**COPD**	**Asthma**	**IPF**	**MLD**
**Total study**		**49**	**23**	**25**	**6**
Actin, cytoplasmic 1	ACTB	Up (11) Down (6)	Up (2) Down (5)	Up (8) Down (3)	Up (2) Down (2)
Alpha-2-HS-glycoprotein	AHSG	Up (2)	Up (1) Down (1)	Up (1) Down (1)	Unknown
Albumin	ALB	Up (11) Down (4)	Up (6) Down (3)	Up (4) Down (4)	Up (2)
Apolipoprotein A-I	APOA1	Up (4) Down (1)	Up (3)	Up (3) Down (3)	Unknown
Apolipoprotein C-III	APOC3	Up (2)	Up (2)	Down (3)	Unknown
Complement C3	C3	Up (6) Down (3)	Up (2) Down (6)	Up (2) Down (10)	Down (2)
Ferritin heavy chain	FTH1	Up (Unknown)	Down (2)	Up (2)	Down (1)
Glyceraldehyde-3-phosphate dehydrogenase	GAPDH	Up (3) Down (4)	Up (2) Down (3)	Up (5) Down (2)	Up (1)
Vitamin D-binding protein	GC	Up (2) Down (1)	Up (3)	Down (4) Up (1)	Up (3)
Glutathione S-transferase P	GSTP1	Up (2) Down (5)	Up (1) Down (5)	Up (0.3) Down (4)	Not changed
Haptoglobin	HP	Up (10) Down (2)	Up (5) Down (2)	Up (7) Down (3)	Up (5)
Heat shock protein beta-1	HSPB1	Up (1) Down (2)	Up (2)	Up (2) Down (2)	Up (1)
Immunoglobulin kappa constant	IGKC	Up (6) Down (1)	Up (1)	Up (4)	Up (1)
Keratin, type I cytoskeletal 10	KRT10	Up (1) Down (3)	Up (1)	Up (1) Down (1)	Up (1)
Keratin, type I cytoskeletal 9	KRT9	Up (2) Down (2)	Up (1)	Down (1)	Up (1)
Lipocalin-1	LCN1	Up (2) Down (1)	Down (2)	Down (1)	Down (1)
Proteasome subunit alpha type-2	PSMA2	Up (1)	Down (1)	Up (1) Down (2)	Down (1)
Retinol-binding protein 4	RBP4	Up (1) Down (2)	Down (2)	Up (3) Down (2)	Unknown
Protein S100-A8	S100A8	Up (8)	Up (4) Down (2)	Up (2)	Up (2)
Protein S100-A9	S100A9	Up (6) Down (3)	Up (5) Down (Unknown)	Up (3) Down (1)	Up (1)
Serotransferrin	TF	Up (2)	Up (1) Down (4)	Up (3) Down (2)	Down (1)
Ubiquitin-conjugating enzyme E2 N	UBE2N	Up (3)	Down (2)	Up (2) Down (1)	Down (1)

### Gene ontology and enrichment analysis

Gene enrichment analysis is a method for analyzing molecular and biological processes between diseases. In addition, gene ontology evaluates biological processes, cellular components, and molecular functions using the FunRich software. This tool identifies GO and biological pathways between shared disease genes. *P*-value < 0.05 was considered as a standard metric for high-score biological pathways.

### Evaluation of hub proteins

**Fig. 5:**
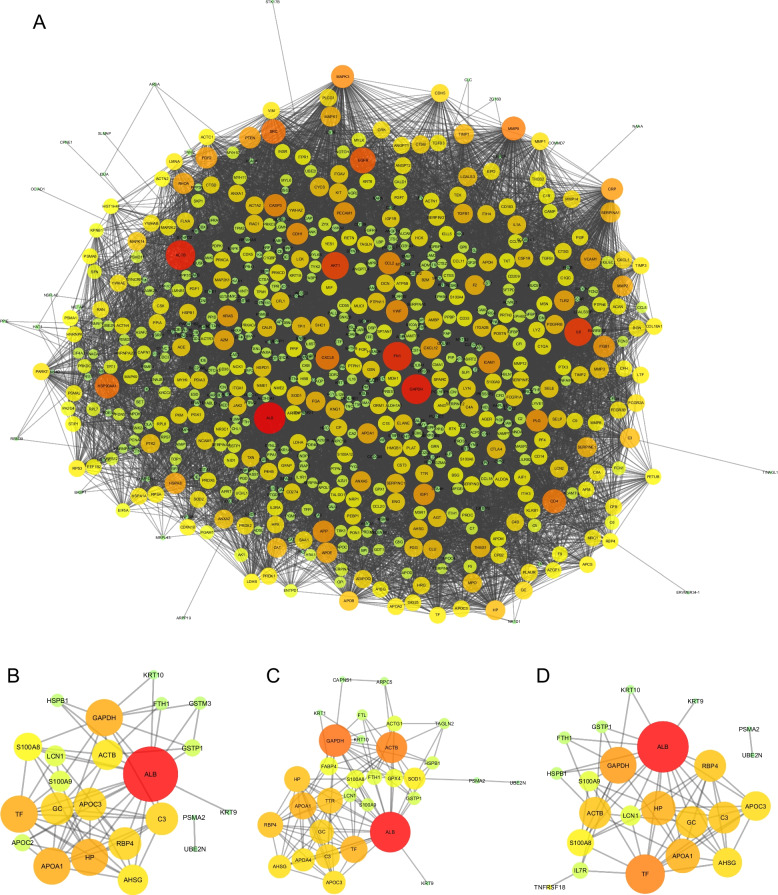
Scheme of shared proteins in diseases. **A** proteins shared by COPD, asthma and IPF, **B** proteins shared by COPD, asthma and MLD, **C** proteins shared by COPD, IPF and MLD, **D** proteins shared by IPF, asthma, and MLD. The size and color of the nodes correspond to the number of interactions they have with other proteins. Thus, a node with a larger size and a redder color indicates more interactions and scores with other proteins, whereas a smaller size and a greener color indicate a lower degree of that node

The PPI network was determined using STRING. Then, using Cytoscape, the molecular pathways and interactions between the shared genes were drawn. The PPI network derived from the shared genes was displayed as nodes and edges based on the degree of importance and interactions. The genes with a high score were selected as the hub. These hub genes can be known as biomarkers, based on which preventive and therapeutic strategies can be designed (Fig. 5).

## Shared biological process and Reactome pathway

With a more detailed evaluation, most of the genes and molecular pathways involved in GO and the biological process were identified. The main biological processes included neutrophil degranulation, innate immune response, post-translational protein modification, and retina homeostasis, while the main Reactome pathways included neutrophil degranulation, platelet degranulation, post-translational protein phosphorylation, and retinoid metabolism and transport (Table 2).

**Table 2:** Some top biological and Reactome pathway between diseases

Asthma-IPF-MLD					
Biological Process	*P*-value	Bonferroni method	Reactome Pathway	*P*-value	Bonferroni method
Neutrophil degranulation	1.9E-07	0.00233	Neutrophil degranulation	5.49E-06	0.013788
Retina homeostasis	1.72E-11	2.11E-07	Regulation of Insulin-like Growth Factor (IGF) transport and uptake by Insulin-like Growth Factor Binding Proteins (IGFBPs)	6.53E-06	0.016399
Post-translational protein modification	6.4E-06	0.078357	Post-translational protein phosphorylation	3.16E-06	0.007939
Cellular protein metabolic process	1.71E-06	0.021	Platelet degranulation	0.000161	0.403806
COPD-Asthma-IPF					
Biological Process	*P*-value	Bonferroni method	Reactome Pathway	*P*-value	Bonferroni method
Neutrophil degranulation	3.1E-66	3.79E-62	Neutrophil degranulation	1.52E-50	3.83E-47
Signal transduction	1.74E-13	2.13E-09	Platelet degranulation	6.24E-41	1.57E-37
Innate immune response	5.05E-18	6.18E-14	Regulation of insulin-like growth factor (IGF) transport and uptake by insulin-like growth factor binding proteins (IGFBPs)	8.41E-23	2.11E-19
Cell adhesion	3.25E-18	3.98E-14	Post-translational protein phosphorylation	9.12E-17	2.29E-13
COPD-Asthma-MLD					
Biological Process	*P*-value	Bonferroni method	Reactome Pathway	*P*-value	Bonferroni method
Neutrophil degranulation	1.9E-07	0.00233	Neutrophil degranulation	5.49E-06	0.013788
Post-translational protein modification	6.4E-06	0.078357	Regulation of insulin-like growth factor (IGF) transport and uptake by insulin-like growth factor binding proteins (IGFBPs	6.53E-06	0.016399
Retina homeostasis	1.72E-11	2.11E-07	HDL remodeling	3.88E-09	9.76E-06
Cellular protein metabolic process	1.71E-06	0.021	Retinoid metabolism and transport	2.39E-06	0.005999
COPD-IPF-MLD					
Biological Process	*P*-value	Bonferroni method	Reactome Pathway	*P*-value	Bonferroni method
Neutrophil degranulation	4.45E-10	5.45E-06	Neutrophil degranulation	4.7E-08	0.000118
Retina homeostasis	2.11E-16	2.58E-12	Post-translational protein phosphorylation	1.74E-06	0.004372
Cellular protein metabolic process	3.93E-08	0.000481	Regulation of insulin-like growth factor (IGF) transport, and uptake by insulin-like growth factor binding proteins (IGFBPs)	4.12E-06	0.010344
Post-translational protein modification	1.13E-05	0.138488	Retinoid metabolism and transport	4.01E-07	0.001007

## Discussion

Lung disorders are among the most shared diseases and are characterized by impaired lung function. There is a wide range of lung diseases whose clinical symptoms overlap; therefore, it is difficult to distinguish them from one another [17]. Shared genes or pathways that can be used to treat these diseases had not previously been identified [18]. In this study, we first examined the genes and pathways shared by four chronic diseases (COPD, IPF, asthma, and MLD). Based on the systems biology approach and using enrichment analysis, we examined the genes and biological pathways shared by the cited disorders. In this section, we first evaluate the biological pathways, in which 22 genes shared by the four diseases are involved, and then assess the shared biological pathways in triplicate.

Haptoglobin (HP) is a factor involved in hemoglobin metabolism. It has also been shown to be involved in the pathogenesis of chronic lung diseases. CD163 is known as the HP receptor. The interaction between HP and CD163 increases the expression of heme oxygenase-1 (HO-1), which reduces inflammation and oxidative stress [19]. CD163 has also been shown to differentiate macrophage type 2 (M2) which, in turn, decreases inflammation in COPD. Considering the role of the MAPK / cAMP / PI3K / AKT pathway in M2 differentiation, CD163 probably causes M2 differentiation by activating this pathway [20]. In asthma patients, the interaction between HDAC8 / Gal3 increases the CD163 expression; therefore, given the role of CD163 in M2 differentiation, and the role of M2 cells in Th2 differentiation, CD163 in asthma can be considered as a target for the targeted therapy route [21].

S100 A8 / A9 was another factor shared by the four diseases. In COPD, S100A8 plays a cyto-protective role. In other words, PKA / cAMP prevents inflammation and the production of reactive species oxygen (ROS). In COPD, S100A8 is dephosphorylated and degraded by SYVN1, which eventually leads to the apoptosis of lung cells [22]. Some studies have reported that S100A8 activates NF-KB through MUC5AC expression, which ultimately increases NLRP3 activation and inflammatory cytokines’ production [23]. Therefore, S100A8 acts as a double-edged sword in COPD. Identifying the pathways involved in pathogenesis and treatment can be effective for designing treatments.

In IPF, an increase in S100A8 triggers inflammatory cytokines’ production by activating TLR4. Moreover, in asthma, it causes inflammation by activating the PI3K / AKT / MAPK / NF-kB pathway [24]. In asthma, the SERPINB3 / B4 complex increases S100A8 production and inflammation by activating P38 / MAPK. S100A8; in addition to causing inflammation through molecular pathways, it can raise the production of complement C3, which is a component of the inflammatory system [25]. This study found that S100A8 / A9 expression was higher in all four diseases compared to controls; since this protein is involved in inflammation and cellular protection, identifying pathways that lead to cellular protection of S100A8 / A9 against inflammation can be a therapeutic route.

C3 increases cell survival by activating the mTOR pathway. In COPD, an increase in C3 leads to CD46 expression. CD46 activates STAT1 and, eventually, BCL-2 by forming a complex with CD3. BCL-2 expression prevents cell apoptosis. In addition, C3 expression in COPD increases the apoptosis of cytotoxic TCD8 cells, which prevents inflammation [26]. C3 expression is increased in COPD patients; therefore, C3 targeting can be a good treatment strategy. In asthma, an increase in C3 leads to innate lymphoid cell chemotaxis (ILC2), which ultimately raises the production of IL-4 and IL-13 and causes inflammation. C3 also increases the expression of CCL2 and CCL5 chemokines by activating the ERK1,2 / MAPK pathway. The expression of these chemokines leads to mast cell chemotaxis and inflammation. On the other hand, adenosine reduces the chemotaxis of mast cells to inflammation site by inhibiting ERK1,2 / MAPK [27, 28].

In IPF, C3 produces IL-17, which eventually causes pulmonary fibrosis through TGF-β / P38 / MAPK. C3 also causes pulmonary fibrosis due to MUC5B expression. Furthermore, TGF-β has been shown to inhibit C3 by inhibiting CD46 and CD55. Therefore, TGF-β, despite causing pulmonary fibrosis in IPF, can prevent inflammation and pulmonary fibrosis in patients by reducing C3 production [29–31]. MLD have also been found to cause C3 inflammation and disease progression and, ultimately, it reduces patient survival [32]. Since C3 expression is decreased in asthma, IPF, and MLD, identifying pathways that reduce its expression can be effective in treatment.

Apolipoprotein A1 (APO A1) is another factor with a dual role; it plays an effective role in the progression and prevention of lung damage due to inflammation by regulating different pathways. In COPD, APOA1 reduces ROS production by NADPH oxidase and NOX3 expression [33]. It also inhibits apoptosis in lung cells by inhibiting NF-κB and Caspase8. In asthma, APO A1 expression inhibits inflammation by inhibiting ERK / NF-κB. It also prevents lung damage and dysfunction by expressing Lipoxin A4 (LXA4) [33, 34]. Moreover, it reduces the chemotaxis of neutrophils to the inflammation site by lowering the VCAM-1 and CXCL5 expression. Ultimately, it decreases TGF-β production [35]. Decreased TGF-β can reduce Th2 differentiation and produce IL-4 and IL-13. In IPF, APO A1 lowers the TGF-β production. TGF-β causes pulmonary fibrosis in patients through the ERK / MAPK pathway. It also reduces M2 differentiation and TGF-β production by decreasing the IL-4 production [36, 37]. Since APOA1 has an anti-inflammatory role and, in this study, its expression had increased in some diseases, identifying the pathways leading to its increased expression can contribute to designing therapeutic methods to prevent inflammation.

CD74 is known as transferrin receptor (TF). TF is involved in the regulation of inflammation and ROS production due to iron metabolism. In COPD, CD74 inhibits macrophage migration inhibitory factor (MIF) expression. MIF generates ROS via the ASK1 / P38 / XOR pathway [38]. CD74 hinders lung cell apoptosis by inhibiting P53 and activating the ERK / MAPK / AKT pathway [38]. Inhibition of MIF expression by reducing NF-κB inhibition and M2 cell differentiation alleviates inflammation [39]. In asthma, MIF induces CCl2, CXCR2, and CXCR4 expression through the ERK / MAPK / P38 / Rho A GTPase pathway. The expression of this chemokine causes the chemotaxis of immune cells and inflammation in patients [40].

Heat shock protein (HSP) has been shown to inhibit MIF. In COPD, increased HSP expression inhibits JNK / NF-κB and prevents inflammation. HSP has also been shown to increase inflammation through the TLR4 / MAPK / NF-kB pathway [41]. Activation of the MMK3 / P38 / NF-kB / Rel A pathway also raises the HSP expression [42]. In asthma, HMGB1 expression induces HSP expression through the TLR4 / MYD88 / NF-kB pathway, which eventually produces IL-4 and IL-13. The generation of these cytokines leads to Th2 differentiation and disease progression [43, 44]. Other studies have reported that HSP induces immune cell chemotaxis and inflammation through the ERK / MAPK pathway. In IPF, HSP produces TGF-β, which causes epithelial mesenchymal transition (EMT) and pulmonary fibrosis through SMAD / P38 / ERK / MAPK [45]. HSP also activates HFL-1 after binding to LRP-1 and eventually produces TGF-β [46].

In mustard victims, as in IPF, HSP causes inflammation through TGF-β and activation of P38 / MAPK pathway [47]. CD74 is a TF receptor, and this protein along with HSP played a dual role in pathogenesis; moreover, the findings of the present study have shown that their expression has variations. Therefore, identifying the pathways that reduce inflammation via HSP and CD47 can contribute to therapeutic designs.

In addition to shared genes, several genes were observed in disease alone and were evaluated under the influence of molecular mechanisms involved in pathogenesis.

### Growth factor receptor-bound protein 2 (GRB2)

GRB2 is a gene expressed on many cells. It binds to several receptors through its domains, including EGFR and FGFR, and regulates many cellular molecular processes. Studies have shown that in COPD GRB2, by activating the PI3K / AKT pathway, it increases the BCL-2 expression and prevents the apoptosis of airway epithelial cells (AECs) [48]. By activating inflammatory cells, it also induces inflammatory reactions in patients. GRB2 has been shown to produce IL-1, IL-6 and TNF-α through MAPK signaling. The production of these cytokines stimulates monocytes and neutrophils and generates inflammatory mediators. GRB2 also produces MMPs and stimulates inflammatory responses in AECs by generating VEGF and activating the MAPK / ERK pathway [48, 49].

The role of GRB2 in the pathogenesis of IPF varies. Accordingly, it has been determined that GRB2 is located downstream of the TGF-β receptor and activates the Raf / MEK / ER1 / 2 pathway. Activation of this pathway leads to the proliferation of fibroblast cells. The activation of the said pathway also stimulates the production of MMPs and the proliferation of collagen cells, which cause fibroblast cells to proliferate [50].

Thus, although GRB2 is involved in both diseases, its downstream pathways differ in each disease, a finding that can help the design of treatment strategies.

### Estrogen receptor 1 (ESR1)

ESR1 was identified as the gene most closely associated with other genes in asthma. ESR1 in asthma regulates the proliferation and remodeling of airway smooth muscle (ASM). The results revealed that ESR1 produces TNF-α and activates PDFG. These factors lead to the production of MMPs and prevent the activation of their inhibitors (TIMPs) [51]. Based on the literature, ESR1 produces TNF-α by activating the NF-κB pathway. The generation of these factors leads to extracellular matrix (ECM) stimulation, which increases the remodeling of ASM. Therefore, the regulation of ESR1 expression in patients can be a therapeutic route to prevent the progression of the disease through targeted therapy [51].

### Interferon Regulatory Factor 7 (IRF7)

To date, no study has been conducted on the molecular mechanism of IRF7 in MLD patients. However, its expression has been shown to decline in these patients compared with healthy individuals. IRF7 is known to suppress inflammation. It reduces the production of inflammatory mediators by suppressing the NF-κB pathway. Thus, since inflammation is an inflammatory mechanism in MLD, the regulation of IRF7 expression can be a suitable treatment route [52, 53].

## Conclusion

The genes and pathways shared by COPD, asthma, IPF, and MLD were investigated. The results showed 22 shared genes involved in many pathways, including lipid metabolism, post-translational protein modification, platelet degranulation, etc. However, most genes were involved in stimulating the immune system and causing inflammation. The dysregulation of proteins can activate signaling pathways and release inflammatory mediators. Still, some of these genes were shown to act as double-edged swords as they both induce and inhibit inflammation. Given that these genes activate different pathways in the cited diseases, identifying the factors and their downstream pathways can be effective in designing preventive and therapeutic strategies.

## Supplementary Information

**Additional file 1.**

**Additional file 2.**

**Additional file 3.**

## Data Availability

The datasets generated and/or analyzed during the current study are available from the corresponding author on reasonable request.
