# Supplementary material for: A computational approach for the identification of key genes and biological pathways of chronic lung diseases: a systems biology approach
Source: BMC Med Genomics. 2023 Jul 8;16:159. doi: 10.1186/s12920-023-01596-7 (PMC10329352; doi:10.1186/s12920-023-01596-7)
Supplement: Supplementary file 2 — Additional file 2. [file 12920_2023_1596_MOESM2_ESM.pptx]

## Slide 1
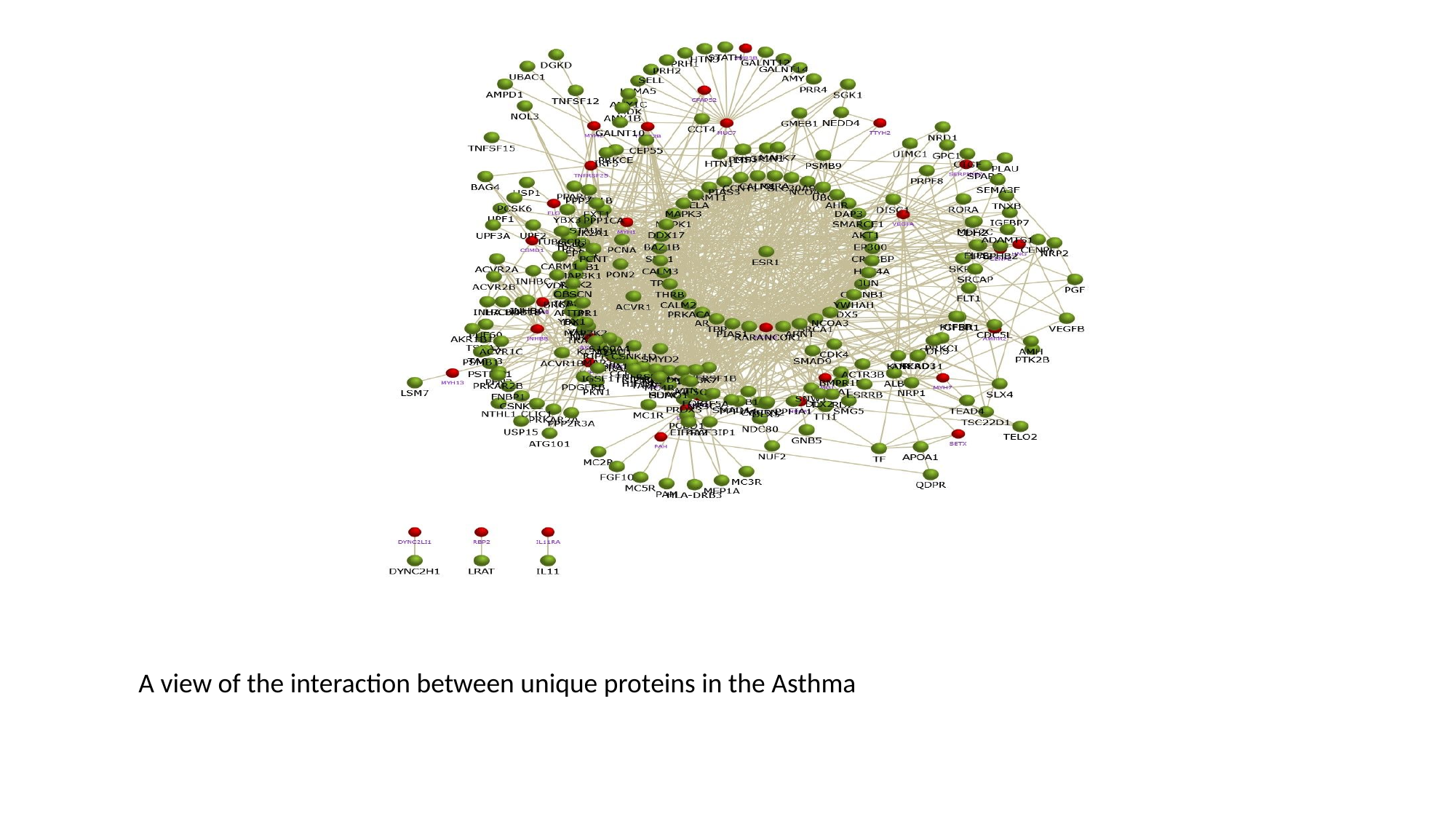

A view of the interaction between unique proteins in the Asthma

## Slide 2
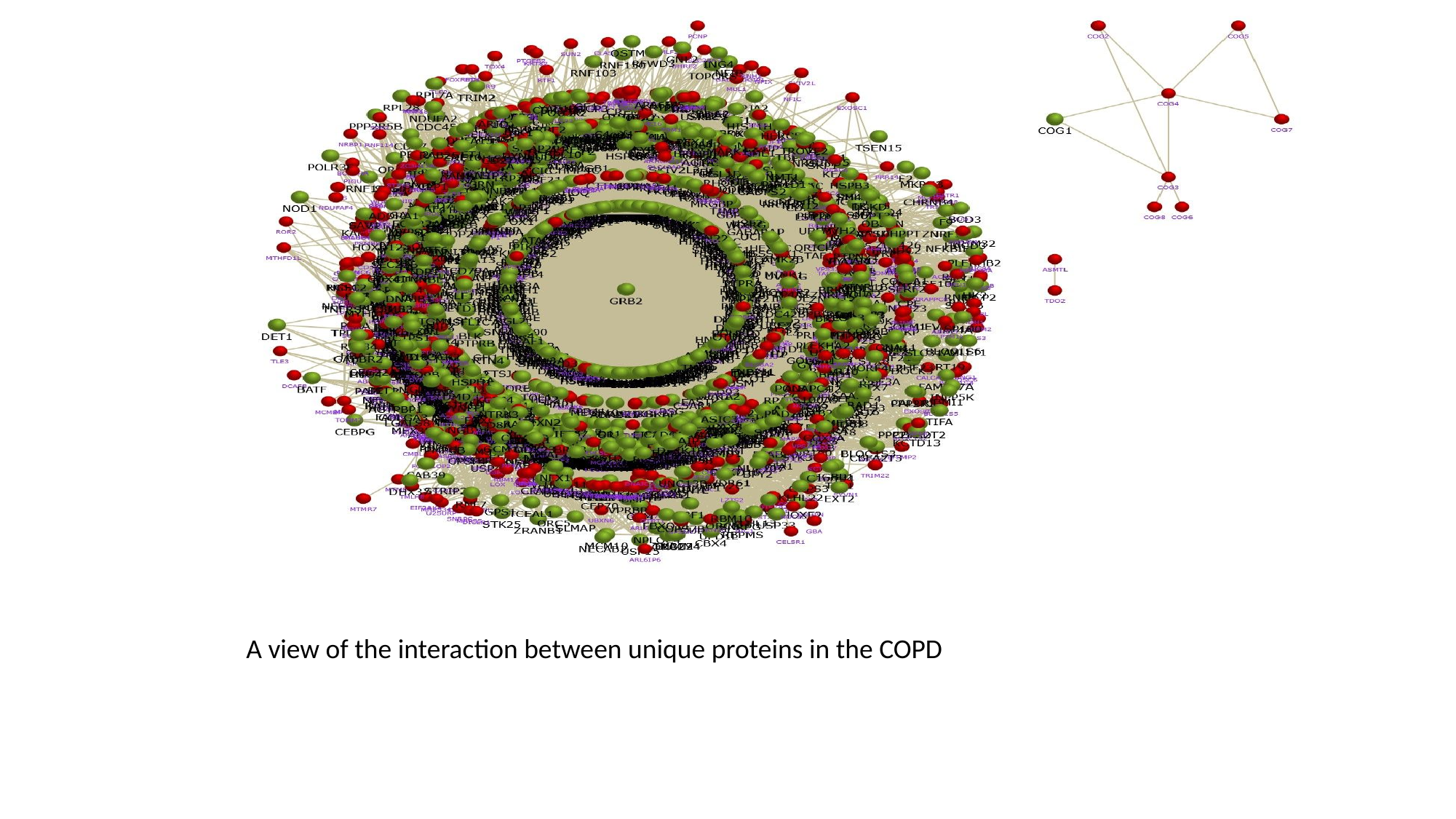

A view of the interaction between unique proteins in the COPD

## Slide 3
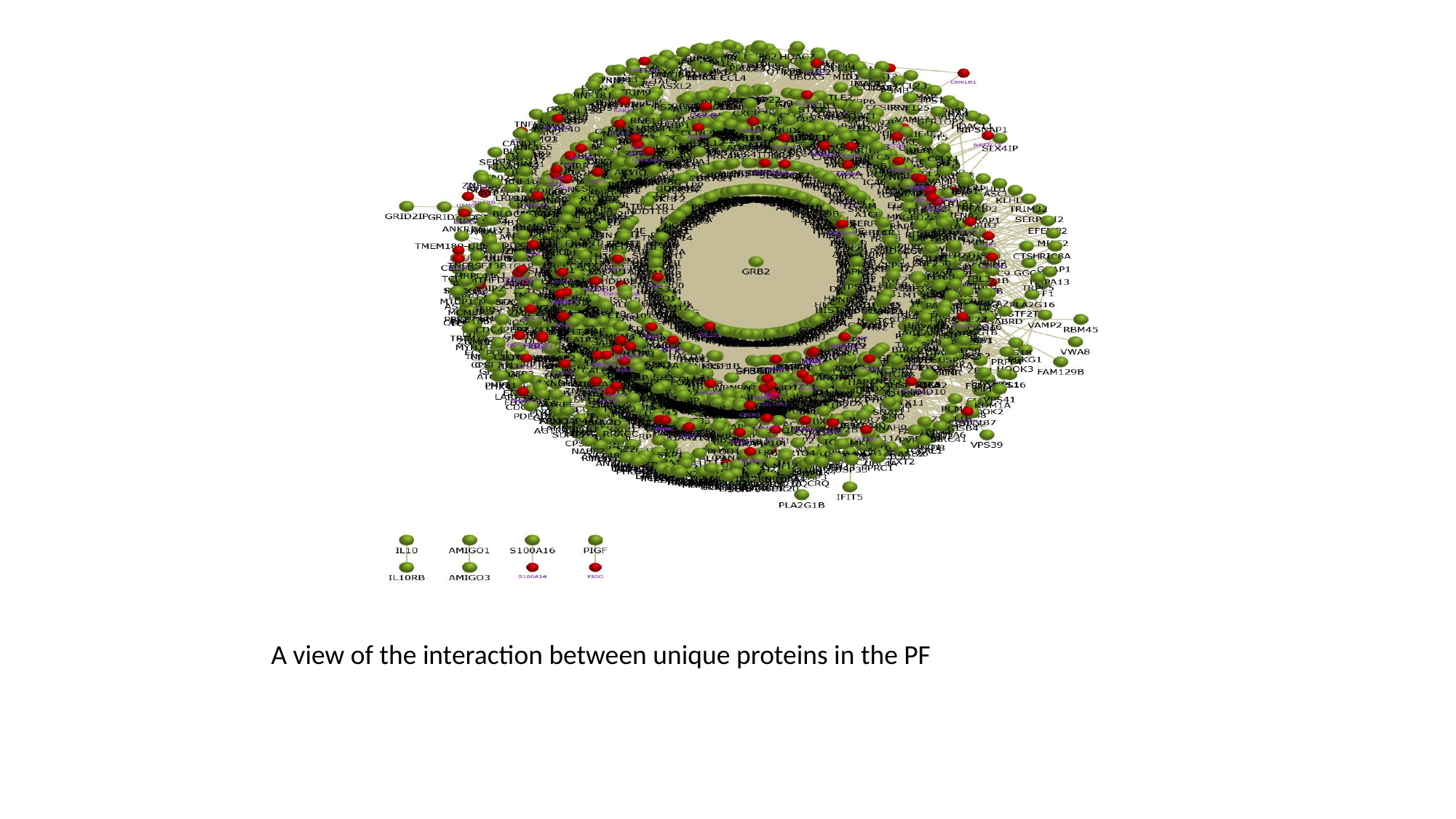

A view of the interaction between unique proteins in the PF

## Slide 4
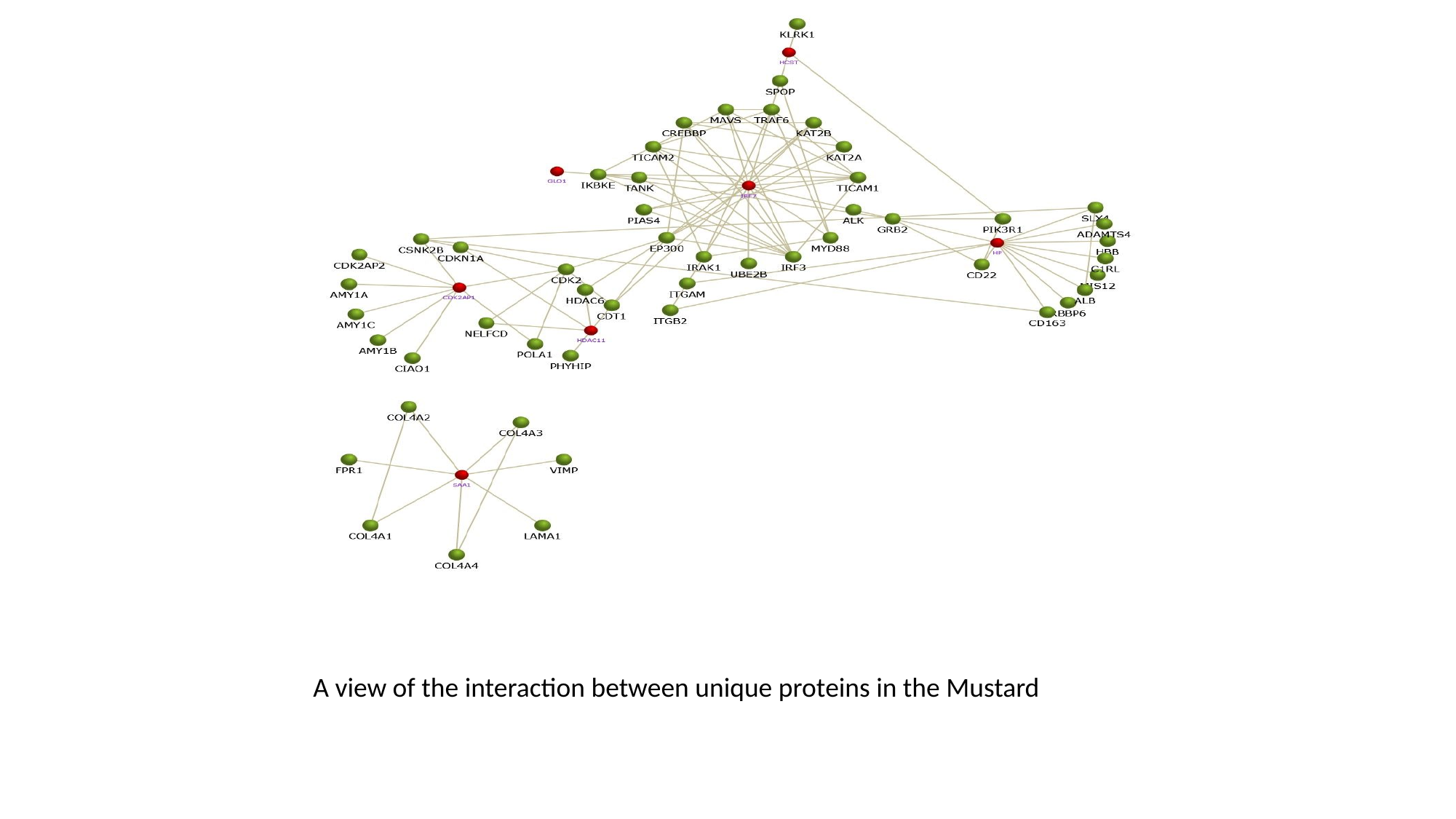

A view of the interaction between unique proteins in the Mustard
